# Supplementary material for: Long Non-Coding RNA LOC401312 Induces Radiosensitivity Through Upregulation of CPS1 in Non-Small Cell Lung Cancer
Source: Int J Mol Sci. 2025 Jun 19;26(12):5865. doi: 10.3390/ijms26125865 (PMC12193141; doi:10.3390/ijms26125865)
Supplement: Supplementary file 1 [file ijms-26-05865-s001.zip › Supplementary Figures.pdf]

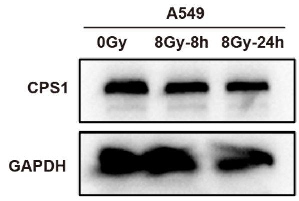

Figure S1. Western blot analysis of CPS1 protein levels in A549 cells at 8 and 24 hours post-ionizing radiation.

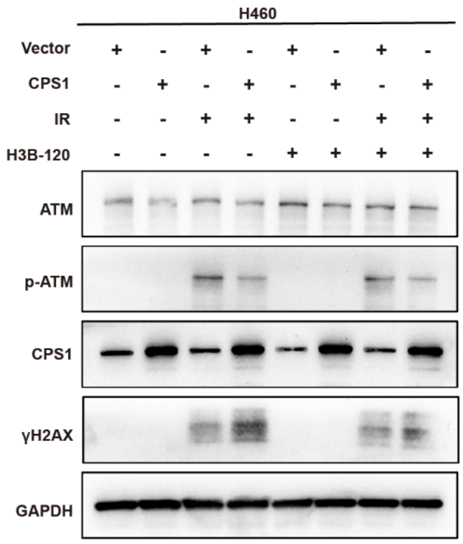

Figure S2. H460 cells were pretreated with 10 μM CPS1 inhibitor H3B-120 for 48 hours preceding 8 Gy ionizing radiation exposure, with protein lysates harvested at 2h post-irradiation and subjected to Western blot analysis.

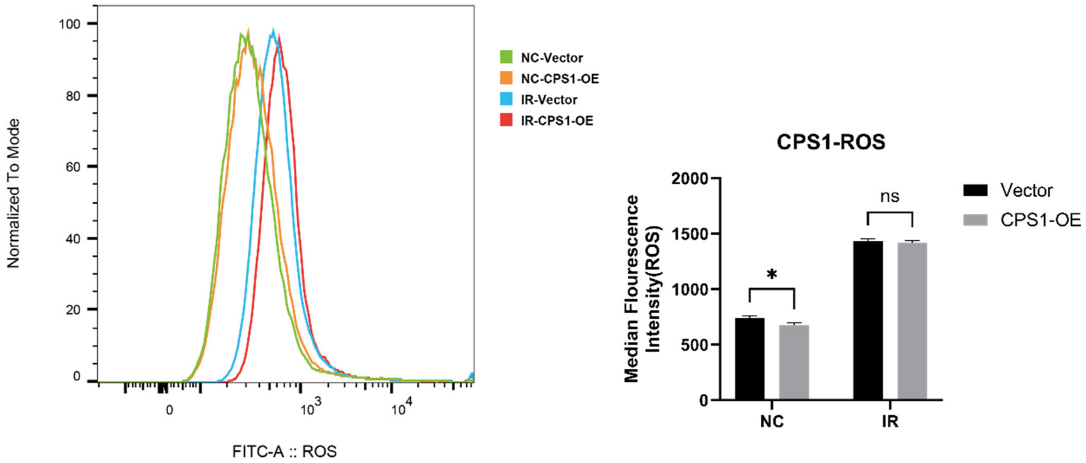

Figure S3. Flow cytometric quantification of ROS levels using DCFH-DA fluorescent probe in CPS1-overexpressing A549 cells at 4 hours post-8 Gy ionizing radiation. n=3,

* $p < 0.1$ by two-way ANOVA.

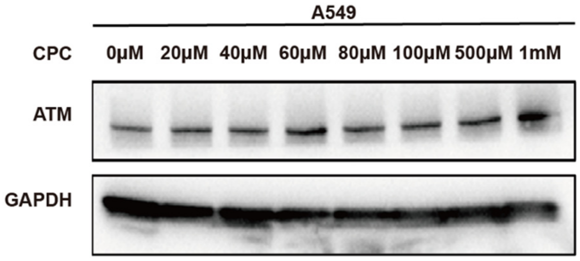

Figure S4. Western blot analysis of ATM protein levels in A549 cells treated with varying final concentrations of carbamyl phosphate disodium salt (CPC) for 72 hours.
